# A Cardiovascular Health and Wellness Mobile Health Intervention Among Church-Going African Americans: Formative Evaluation of the FAITH! App

**DOI:** 10.2196/21450

**Published:** 2020-11-17

**Authors:** LaPrincess C Brewer, Ashok Kumbamu, Christina Smith, Sarah Jenkins, Clarence Jones, Sharonne N Hayes, Lora Burke, Lisa A Cooper, Christi A Patten

**Affiliations:** 1 Department of Cardiovascular Medicine Mayo Clinic Rochester, MN United States; 2 Center for Healthy Equity and Community Engagement Research Mayo Clinic Rochester, MN United States; 3 Robert D and Patricia E Kern Center for the Science of Health Care Delivery Mayo Clinic Rochester, MN United States; 4 Division of Health Care Policy and Research Mayo Clinic Rochester, MN United States; 5 Division of Biomedical Statistics and Informatics Mayo Clinic Rochester, MN United States; 6 Hue-MAN Partnership Minneapolis, MN United States; 7 School of Nursing Department of Health and Community Systems University of Pittsburgh Pittsburgh, PA United States; 8 Department of Health Behavior and Society Johns Hopkins Bloomberg School of Public Health Baltimore, MD United States; 9 Department of Medicine Johns Hopkins School of Medicine Baltimore, MD United States; 10 Department of Psychiatry and Psychology Mayo Clinic Rochester, MN United States

**Keywords:** mobile health, eHealth, community-based participatory research, health promotion, African Americans, mobile phone

## Abstract

**Background:**

In light of the scarcity of culturally tailored mobile health (mHealth) lifestyle interventions for African Americans, we designed and pilot tested the Fostering African-American Improvement in Total Health (FAITH!) App in a community-based participatory research partnership with African American churches to promote cardiovascular health and wellness in this population.

**Objective:**

This report presents the results of a formative evaluation of the FAITH! App from participants in an intervention pilot study.

**Methods:**

We included 2 semistructured focus groups (n=4 and n=5) to explore participants’ views on app functionality, utility, and satisfaction as well as its impact on healthy lifestyle change. Sessions were audio-recorded and transcribed verbatim, and qualitative data were analyzed by using general inductive analysis to generate themes.

**Results:**

In total, 6 overarching themes emerged among the 9 participants: overall impression, content usefulness, formatting, implementation, impact, and suggestions for improvement. Underpinning the themes was a high level of agreement that the intervention facilitated healthy behavioral change through cultural tailoring, multimedia education modules, and social networking. Suggestions for improvement were streamlining the app self-monitoring features, prompts to encourage app use, and personalization based on individuals’ cardiovascular risk.

**Conclusions:**

This formative evaluation found that the FAITH! App had high reported satisfaction and impact on the health-promoting behaviors of African Americans, thereby improving their overall cardiovascular health. Further development and testing of the app among African Americans is warranted.

**Trial Registration:**

ClinicalTrials.gov NCT03084822; https://clinicaltrials.gov/ct2/show/NCT03084822.

## Introduction

Ideal cardiovascular (CV) health has been defined by the American Heart Association (AHA) as the achievement of optimal levels of 7 key components, including health-promoting behaviors (optimal diet and physical activity [PA], achieving normal weight, and abstaining from smoking) and controlling health factors (blood pressure, cholesterol, and blood glucose). Each of these components is independently associated with lower CV disease risk in a stepwise fashion; however, there are striking racial and ethnic disparities in ideal CV health [1]. Compared with White Americans, African Americans (AAs) have a lower prevalence of ≥5 ideal components (11.8% vs 19.2%) [2] and an 82% lower likelihood of achieving ≥5 of the 7 ideal components [3]. Furthermore, it has been estimated that the components with the greatest potential for improvement in AAs are health behaviors, including diet, PA, and weight management [4]. Even modest shifts in the distribution toward improved CV health in the AA population could result in appreciable reductions in incident CV disease in this group. Thus, innovative, individual, and population-based interventions to promote ideal CV health through lifestyle changes among AAs are desperately needed.

Mobile health (mHealth) [5] lifestyle interventions hold potential for diffusing CV health promotion in AA communities. AAs are embracing mobile technologies with rapid smartphone use expansion and frequent internet searches for CV disease–related health information [6] and positively view mHealth interventions as viable strategies to improve health outcomes [7,8]. However, only a handful of studies have tested mHealth interventions to improve CV health in AAs, with many studies focusing only on health behaviors (eg, diet, PA, weight loss) or single chronic medical conditions (eg, diabetes, hypertension, heart failure) [9-17].

Emboldened by the AHA 2020 Impact Goals to improve the overall CV health of AAs, we co-designed a culturally tailored digital app, Fostering African-American Improvement in Total Health App (FAITH! App), to promote CV health within the AA faith community using an iterative participatory approach within an established academic-community partnership [18,19]. The intervention is grounded in behavioral theoretical frameworks, including principles from the health belief model [20], social cognitive theory [21], and the community mobilization model [22,23]. At the heart of the intervention are multimedia education modules that were guided in design by the health belief model, which incorporates an individual’s perceived susceptibility, benefits, and barriers to predict behavior change. The social cognitive theory model, or learning through a collective agency to influence health behaviors, was incorporated in specific app features (eg, group sharing board, testimonials on healthy lifestyle) as well as our inclusion of an established social construct within the AA community, the Black Church [24]. To this end, we also embraced the community mobilization model by actively engaging in a strategic community partnership while leveraging the norms, values, and resources it embodied to develop and implement an intervention [25].

To our knowledge, our parent study is among the first to combine a culturally tailored and app-based lifestyle intervention to promote ideal CV health within the context of a primary CV disease prevention program [18,19,26]. Participants within our pilot study achieved improvements in key CV health behaviors and factors and reported overall high acceptability and satisfaction of the intervention [18,26]. To address the dearth of literature integrating formative processes to design and evaluate culturally tailored mHealth interventions, we used a qualitative approach to further explore participant experiences of taking part in the intervention.

This program evaluation aims to gain insight into the impact of an mHealth lifestyle intervention (FAITH! App) on the CV health behaviors of AAs participating in a quasiexperimental behavioral intervention pilot study. We sought to include participants’ perspectives to evaluate the intervention with the goal of integrating this information in future iterations of our mHealth intervention to more effectively influence CV health in this population.

## Methods

The parent study was approved by the Mayo Clinic Institutional Review Board and registered (ClinicalTrials.gov [NCT03084822]), and participants provided written informed consent before participation.

### Research Design and Participant Recruitment

The parent study comprised 3 community-driven phases: (1) app design with the AA community through formative app development [18], (2) app pilot testing [26], and (3) app evaluation using quantitative and qualitative research methods. This analysis (phase 3) evaluates qualitative data on participants’ perceptions of app pilot testing, who were subsequently recruited to participate in the evaluation component of our study. Details on the parent study rationale, recruitment procedures, and participant inclusion/exclusion criteria for the overarching parent study have been described [27]. Briefly, we collaborated with 5 predominately AA churches in Rochester and Minneapolis-St Paul, Minnesota, using a community-based participatory research (CBPR) approach to co-design a CV health and wellness digital app–based program (ie, the FAITH! App) [18]. A total of 50 AA church parishioners were enrolled into a single-group pilot study to follow a 10-week intervention centered on the FAITH! App. Its components included 10 core multimedia education modules delivered by health professionals on CV health, interactive diet and PA self-monitoring, and social networking through a group sharing board. Participants completed health assessments, including self-administered electronic surveys of sociodemographic and health behavior information as well as physical examinations and laboratory studies of biometrics at baseline and 28 weeks postintervention. Following the final health assessment, participants from the pilot study were invited to participate in the postintervention focus groups by email. Participants involved in the co-design of the FAITH! App were not invited to participate in the focus groups. There was a 32% response rate (16 out of 50 pilot study participants) to the email invitation. Ultimately, 9 participants were able to participate on the most convenient dates and times chosen by a polling of the interested invitees. Participants received a US $50 cash card for participation in the focus groups.

### Data Collection

Two separate focus groups (N=9) were held to assess participant perceptions of the FAITH! App (one each in Rochester, Minnesota [focus group 1: n=4], and Minneapolis-St Paul [focus group 2: n=5]). Participants’ sociodemographic information, including age, sex, marital status, education level, employment status, and eHealth literacy (measured by the eHealth literacy scale [eHEALS], an assessment of an individual’s perception of their ability to understand and apply electronic health information) [28-30], were derived from baseline electronic surveys completed at the time of pilot study enrollment.

We developed a comprehensive moderator guide inclusive of neutrally worded, open-ended questions to facilitate open discussion and to obtain feedback on the FAITH! App intervention including the following core topics: app satisfaction, dislikes, and suggestions for improvement. The moderator guide was developed from a literature review [31], our participatory intervention design process [18], informal discussions with participants and church partners as well as quantitative evaluations of the FAITH! App. The primary moderator (CS) had substantial experience conducting focus groups among racial and ethnic minority populations and previously collaborated with the study team during the formative design of the FAITH! App with a similar group of AAs [18]. The study principal investigator (LB) was intentionally not present during the focus groups to minimize social desirability bias and coercion among the study participants. However, the moderator and study principal investigator met before each focus group to review specific strategies to encourage group discussion and culturally tailor questions to place the group at ease to share their valued opinions. Each focus group was 90 min and audio-recorded. The focus group in Rochester was held in a conference room in an outpatient clinical practice in the evening. The Minneapolis-St Paul focus group took place in the evening in a conference room at a community center. Each focus group commenced with a friendly *ice-breaker* to promote sharing among the group, “What is your favorite thing about Minnesota?” Following each focus group, the moderator compiled summary analysis notes of the most salient participant responses, which were then reviewed by the study principal investigator. All sessions were subsequently transcribed verbatim by an experienced transcriptionist.

### Data Analysis

For qualitative analyses, the first author (LB) and a trained qualitative methods specialist (AK) first read the transcripts several times with reference to the focus group moderator guide. The 2 coders collaboratively developed a code book in an iterative process to better organize and categorize the data in accordance with the open coding method, a process that enhances the probability of garnering novel insights [32]. In the code book, each code was described with a concrete definition, and at least one example quote from the data was included. QSR NVivo software version 10 was utilized to organize and manage transcribed data from the focus group sessions. General inductive analysis was used as it aligned with the exploratory and formative nature of the study in the following ways: (1) it condenses raw textual data into a more succinct summary; (2) it links the evaluation (in our case of an intervention) to the summary findings from the raw data; and (3) it cultivates a framework of the underlying structure of experiences or processes evident in the raw data [33]. Two coders (LB and AK) coded all transcripts independently and assigned codes for subsequent categorization around salient themes. To mitigate interpretative biases and to maintain the consistency of coding, the 2 coders met regularly and discussed all coded data until they reached a consensus. Whenever the 2 coders had difficulty assigning a common code, a third team member (CP) assisted with resolving discrepancies or assigned new codes to ensure consensus. Through this collaborative process, the team attained very good agreement on coding [34]. While ensuring intercoder reliability, the 2 coders discussed the main themes in detail, and provisional subthemes also emerged from the coded data [35]. Finally, to further enhance reliability and as a part of our CBPR approach to formative evaluation [18], we reviewed the compiled themes/subthemes with community church partners for member checking and feedback on the themes. We provided the 4 church partners with a preliminary summary of the main themes and subthemes for their review. We then organized a 1-hour teleconference to discuss the preliminary summary (community member checking) among the group with the study principal investigator (LB) and qualitative research expert (AK) moderating. The church partners were in overall agreement with the themes/subthemes and felt that they were in concordance with the general feedback they informally received from study participants at their respective churches. This triangulated process of integrating input from participants, church partners, and the study team provided an additional layer of corroboration to the finalized main themes and subthemes [36,37]. Quotations from participants were extracted from transcripts that best represented participants’ experiences. Descriptive analyses were completed for all sociodemographic data with the calculation of frequencies and proportions. Participants were categorized by app frequency of use as follows: high frequency users (viewing at least 50% of the 10 education modules, tracking at least weekly diet/PA, and posting on the sharing board at least once per month) and low frequency users (those not meeting high frequency usage patterns). All quantitative analyses (demographic data) were conducted in 2017 with SAS version 9.4 (SAS Institute Inc).

## Results

The results of the quantitative survey revealed that focus group participants (N=9) had a mean age of 47.9 years (SD 12.1; median 49.0), were primarily women (6/9, 67%), reported an education level of some college (5/9, 56%), and were mostly employed (8/9, 89%; Table 1).

The average eHealth literacy score was 31.7 (high range by eHEALS ≥26); 7 of 9 participants were classified as high frequency app users. Overall, participants in the Rochester and Minneapolis-St Paul focus groups had similar responses as the vast majority of the generated codes were mentioned by both groups. Thus, data were pooled for both focus groups. Findings of the focus group discussions were grouped into 6 main themes that were divided into corresponding subthemes: (1) overall impression, (2) content usefulness, (3) formatting, (4) implementation, (5) impact, and (6) suggestions for improvement (Table 2).

We present the results by starting with the participants’ global perceptions of the FAITH! App that encompasses their overall acceptability and utility of the intervention. We then examine more granular insights regarding app content and delivery modalities, which could inform future iterations of the intervention. Next, we move to the app’s impact by highlighting its influence at the individual, interpersonal, and community levels. Finally, we conclude with feedback for app enhancement.

**Table 1:** Sample characteristics (N=9).

ID	Age (years)	Sex	Marital status	Employed, at least part-time	Education level	eHealth literacy score	App use frequency
1	26	Male	Married	Yes	Some college	36	Low
2	57	Female	Single	Yes	Technical or associate’s degree	31	High
3	64	Female	Divorced	Yes	College graduate	32	Low
4	52	Female	Committed relationship	No	Some college	27	High
5	43	Male	Married	Yes	Some college	37	High
6	60	Male	Married	Yes	Some college	25	High
7	49	Female	Married	Yes	College graduate	30	High
8	44	Female	Widowed	Yes	Some college	31	High
9	36	Female	Divorced	Yes	Technical or associate’s degree	36	High

**Table 2:** Core themes and subthemes.

Themes		Subthemes
**Theme 1: Overall impression**		Promotion of healthy behaviors by self-monitoring Accessible and instructional health content Influence on healthy lifestyle adoption of the church Technical difficulties with self-monitoring
**Theme 2: Content usefulness**		
	Education modules: General	Thought-provoking and self-reflective Descriptive on cardiovascular health Easy to comprehend health information
	Education modules: Videos	Engaging presentations Convenient and succinct Effective visual learning
	Education modules: Premodule and postmodule self-assessments	Complementary to video series
	Education modules: Brochure content	Reinforcement and summary tool Time-consuming Technical difficulties with access
	Recipes	Inspired action toward healthy eating Better understanding of benefits of healthy eating
	Sharing board	Motivational to healthy lifestyle change Support network Information exchange between participants
	Testimonials	Preferred heart disease survivors or those making healthy lifestyle changes
	Self-monitoring (diet and physical activity)	Fostered personal accountability toward a healthy lifestyle
**Theme 3: App format**		
	General	Fulfilled expectations for continued engagement Diversity of health care professionals within the education modules
	Attentiveness to nuanced cultural perspectives	Importance of linking faith to health for the African American community Cardiovascular health disparities affecting African Americans Implications of visual representations of African Americans
**Theme 4: App implementation**		
	Facilitators for use	Positive messaging supported engagement Focus on benefits of healthy lifestyle Simple navigation Visual display of education module progress Education module variety of activities appealed to differing learning styles Overview of cardiovascular risk factors at start of education modules Consolidation of cardiovascular health information through mobile technology
	Barriers to use	Cumbersome data entry and log-in process Inability to download app on other personal mobile devices
**Theme 5: App impact**		Positive changes to dietary patterns Better awareness of long-term benefits and motivation to make healthy lifestyle changes Team-based lifestyle changes among couples and across generations Positive influence on the patient-provider relationship Health promotion within the church congregation
**Theme 6: Suggestions for app improvement**		Visuals to see progress of diet and physical activity self-monitoring/tracking Automatic syncing function from other diet and physical activity apps Additional functions for education modules and sharing board Individual tailoring of the app to encourage app use and increase its relevance

### Theme 1: Overall Impression

The focus groups opened with questions about the impressions of the app as a whole. The most common positive perceptions identified by the participants were related to health promotion through the easily accessible health information delivered by the education modules. The most common source of challenges with app use came from technical difficulties with the diet and PA self-monitoring feature.

#### Promotion of Healthy Behaviors by Self-Monitoring

Participants saw great value of the app self-monitoring features as they compelled them to keep track of their diet and PA patterns. One participant mentioned how this feature provided accountability to meet personal goals to eat healthier and remain active:

And one thing I did really appreciate about the tracking, I never knew how few vegetables I ate. I would walk down to the cafeteria and purposely go get more fruits and more stuff like that...I gotta go work this off...making sure that I’m staying on track.

Focus group 1, participant

#### Accessible and Instructional Health Content

Most participants reported that the app provided easily accessible and useful information and found the pictorial images and visuals to explain the health information helpful:

I did like how it gave you details and pictures of what they were showing you and explaining.

Focus group 2, participant

#### Influence on Healthy Lifestyle Adoption of the Church

A fundamental concept from several participants was that the genuine commitment to improving the health and well-being of the study participants conveyed by the study team inspired a commitment from the church congregations to focus on adopting healthy lifestyles. The app intervention as a whole seemed to awaken the church congregations to create an endurable culture of healthy living even beyond the completion of the study:

It was really needed, and it took you all to awaken us and start to awaken the church of an area where the church had been negligent...when you’re talking about health. This is a life-time commitment that we’re making, not for one year, but you certainly have put that in the congregation to be more healthy…and then because you got the pastors on board, then that would help plan it, and it will keep going. Hopefully, if this come out for everybody to use.

Focus group 2, participant

#### Technical Difficulties With Self-Monitoring

Participants provided shared challenges with technical difficulties with the app self-monitoring feature, specifically troubleshooting to enter their diet and PA information.

### Theme 2: Content Usefulness

Naturally, discussions transitioned to participant perceptions of the core app features. Overall, the participants found the features useful to better understand the impact of CV risk factors on CV physiology and the development of CV disease. They also mentioned that the app provided them with tools to improve their CV health.

#### Education Modules: General

##### Thought-Provoking and Self-Reflective

One participant felt that the app offered a new awareness of their own health and wellness through the information provided:

Even if there was a day I was thinking about my health and the way that I’m taking care of myself, there was information in here that I wouldn’t normally think about, even on a day when I was thinking about it. So I thought the information was excellent.

Focus group 2, participant

##### Descriptive Content on CV Health

Participants appreciated the descriptive content that allowed them to connect health behaviors such as diet as well as psychosocial factors such as stress to heart functioning:

Sometimes you don’t think about how one thing can affect another thing, and so you looked at everything and how...eating too much sodium can affect how your heart pumps and how stress does this. So everything was very informative, gives you some ideas to think about.

Focus group 2, participant

##### Easy to Comprehend Health Information

The participants felt that the information was presented at an optimum level for understanding across different learning styles. They also appreciated that the speakers avoided extensive use of medical jargon but instead relayed information in layman's terms:

...*the speakers were very descriptive; and for me personally, they didn’t get so deep that they was over my head where I didn’t understand...So it wasn’t like it was a doctor speaking to a nursery school person or something. It was right in the ballpark academically as far as understanding the information that was given; and for me, it was excellent!* [Focus group 2, participant]

#### Education Modules: Videos

##### Engaging Presentations

Participants provided positive feedback about the education module video series, describing it as an engaging and interactive tool that prompted further insights into healthy behavior change:

...the speakers, just the way they presented held my attention. I didn’t doze on any of them. I wasn’t, [like] I wish they’d hurry up and get done. I actually was listening to what they were saying and, even taking mental notes about what I could do differently my own self.

Focus group 2, participant

##### Convenient and Succinct

Participants found the video series format to be a convenient and succinct way to view and access health information both independently and with their families. Several shared how they would watch the videos from a variety of locations (eg, home, work) and revisit the modules for reinforcement. Keeping the videos brief and concise was also viewed favorably. Another participant enjoyed having the summary video at the end of each module to tie together the key concepts:

...and the review at the end...to me, that was the best part because it summarized everything.

Focus group 1, participant

##### Effective Visual Learning

Overall, participants enjoyed the videos with visual depictions of normal and abnormal heart physiology. Several participants commented extensively on the usefulness of slide illustrations and graphics integrated by speakers:

...when they was talking about the heart, showing me how my heart works...for a visual person, that was really helpful to see...not just talking but actually showing me what’s happening to my body.

Focus group 2, participant

### Education Modules: Premodule and Postmodule Self-Assessments

#### Complementary to Video Series

Participants felt that the premodule and postmodule self-assessments (quizzes) were a great addition to the modules and complementary to the video series. They unanimously recommended that they should remain mandatory in order to mark completion of the education module as they were beneficial self-assessment tools. The quizzes also provided an infrastructure to remain on track with the education modules:

I thought it was...nice to have that pretest and the posttest, just to keep you on point...

Focus group 2, participant

### Education Modules: Brochure Content

#### Reinforcement and Summary Tool

Some participants found the brochure content within each education module to be a useful resource to summarize information relayed within the video series:

I thought the brochures are always a good way to sum up everything that they’ve talked about. So before doing the last quiz, I’d always like to just go through quick and read those brochures.

Focus group 1, participant

#### Time-Consuming

Although the content was viewed as useful and relevant, some participants found review of this information to be time-consuming and too much to digest as an addition to the other module features:

...at least I didn’t really want to skip any of it, but it was a lot to consume at one time...and this is interesting, so I don’t really want to stop, but it’s taking my time...it was a lot of reading, but it was good, but it was just a lot to digest at once.

Focus group 2, participant

#### Technical Difficulties With Access

Participants noted that access to the brochure content was cumbersome as the content was not housed within the app itself and required redirection to PDF files within a separate screen.

### Recipes

#### Inspired Action Toward Healthy Eating

Several participants enjoyed heart healthy recipes on the app and shared experiences at the individual and church level of how they incited healthy eating. One participant shared how his family modified their traditional holiday practices toward healthy dietary change through smaller portion sizes:

...this past Thanksgiving, me and my wife, we even changed our whole ... traditional Thanksgiving; we did something totally different. We usually get the turkey ...and was going to put it in the oven or deep fry it or however they do it. We did a small turkey breast, a little turkey breast…we just made everything smaller. And then we just did smaller portions…everything was done and we was good. We didn’t throw a lot out. We didn’t waste a lot of money. And we were a lot healthier.

Focus group 2, participant

At the church level, participants held a church potluck to try out recipes in order to introduce each other to healthier options. They wanted to know if they could integrate healthier ingredients into their daily lives. Many enjoyed the dishes as they maintained flavor without added salt or butter:

...so when we did bring it to our church, we all got to taste different recipes, and it was changing the way of looking at life where I don’t have to have all of this salt, ...so it just gave me a new way of looking at different seasonings or butter...it’s a more healthier version of it, but I was like, hey, I could use this. So I do like that and it still had flavor...we still enjoyed it. We had greens that was cooked in different things and they taste good!

Focus group 2, participant

#### Better Understanding of Benefits of Healthy Eating

Participants discussed that eating healthier can sometimes be more expensive, but they felt the value of eating healthier to improve overall health outweighed the extra costs:

...then here’s the thing too,...everything that’s healthy for us, it costs more money. But we waste money on things we don’t need anyway...So why not spend a little money on something that’s going to help our bodies...If you eat a burger for a buck every day...You’ll be going to the hospital for a heart attack; but if you eat a salad every day, it might cost you more, but you’re healthier.

Focus group 2, participant

### Sharing Board

#### Motivational to Healthy Lifestyle Change

Participants used the sharing board posts to connect with others and found the posts to be very encouraging and motivating to maintain healthy behaviors.

One participant shared:

Those...posts that a lot of people made, to me, were very encouraging...kind of like testimonials to a degree, so it helped me...try to get my game back up closer to everybody’s...

Focus group 1, participant

#### Support Network

Participants also found the sharing board to be a support network to better support others through personal struggles. One participant revealed empathy for another participant through prayer:

They would tell what was going on in their life. And so I was actually able to feel for them...I mean, we’re calling this a faith thing, so I would pray for other folks cuz I can see that they’re going through things.

Focus group 2, participant

#### Information Exchange Between Participants

Participants enjoyed sharing and learning from each other through a variety of communication modalities on the sharing board (eg, text, pictures, videos):

...somebody posted a recipe or someone...cuz people were adding pictures...of things that they cooked...and some people posted recipes. It was amazing how many people would say you can find a large bag of green peppers for $2 at XYZ.

Focus group 1, participant

For those who used it less often, they reported that they still enjoyed reviewing the posts and in hindsight wished that all participants would have used the feature more often:

I thought the sharing board was good. I wish I would have used it more, and I wish everybody else would have used it.

Focus group 2, participant

### Testimonials

#### Preferred Heart Disease Survivors or Those Making Healthy Lifestyle Changes

Participants appreciated the testimonials from the church pastors and past FAITH! participants but initially thought they would feature those with an experience of CV disease or those who have made significant lifestyle changes. They felt that hearing stories from them would inspire them to reflect on their own risk for CV disease. One participant shared:

...it might be good to have, people who may have experienced a heart issue and maybe changed their life...and someone that could encourage you to engage. It probably would be more effective to [make one say] “I really need to think about this more.”

Focus group 1, participant

### Self-Monitoring (Diet and PA)

#### Fostered Personal Accountability Toward a Healthy Lifestyle

Overall, participants found the self-monitoring feature useful as it fostered personal accountability toward a healthy lifestyle. One participant shared how the feature helped to “keep track” of diet and PA patterns:

...some of the people really like tracking and they did it religiously...they prefer to track...

to stay true to the expectation of what you were doing, and then you could also see where you were falling short.

Focus group 1, participant

### Theme 3: App Format

#### General

##### Fulfilled Expectations for Continued Engagement

Overall, the participants enjoyed using the app, with several directly expressing that they would want to continue with the digital formatting of the intervention:

We got enough but we want more!

Focus group 2, participant

Participants also felt that the interactive multimedia delivery modality was appropriate with the goal of increasing awareness of healthy lifestyle change:

They were very informative. The way they presented it, it was easy to understand, very insightful, especially for me because this is an area that I really don’t pay attention to...I realized I had to change my diet; and then once I realized that, then I started listening to these apps and these doctors, I really enjoyed them. I really, really enjoyed them; I really did.

Focus group 2, participant

The participants emphasized that the education modules were essential components of the platform. They also found the homepage inviting, colorful, and eye-catching. Arranging simple icons on the homepage was viewed as a great technique to organize module topics:

These little icons, I thought, were very good, too, for the links. You know, like physical activity, you had the little man walking in healthy living.

Focus group 1, participant

##### Diversity of Health Care Professionals Within the Education Modules

Participants also took notice of the diversity of health care professionals included within the education module videos that included multidisciplinary individuals (eg, cardiologists, endocrinologists, nurses, dieticians).

#### Attentiveness to Nuanced Cultural Perspectives

##### Importance of Linking Faith to Health for the AA Community

The participants commended the study team for incorporating biblical scriptures and spiritual messaging within the app as religious involvement is of high importance to AAs and showed humility to this prioritized AA faith community. They appreciated the emphasis on connecting spiritual and physical health for healthy lifestyle changes, as described by this participant:

I really liked the fact that the scriptures and things like that were there because it seemed like, okay we’re tying faith together with healthy living...I thought that was really awesome; and the little faith icon in the corner, it’s a nice touch that was there...

Focus group 1, participant

##### CV Health Disparities Affecting AAs

The vast majority of participants acknowledged the importance of placing emphasis within the modules on the CV health disparities burden among AAs. They felt that the positive messaging strategies were delivered in an appropriate manner to not place offense or blame on this group but to inform them of their elevated risk for CV disease. This was encompassed in a statement from one participant with many nodding in agreement:

...not pushing one culture over another but still letting you know that this culture is more at risk, and I thought they did that, for lack of a better word, very tactfully, very swoopy where you didn’t feel offended or saying that this was speaking against your culture—you people get this and you people are targets for this. I didn’t feel like it was being pointed out, but I also understood that you were being addressed.

Focus group 2, participant

##### Implications of Visual Representations of AAs

In general, the group felt that the selected photographs of AAs included in the app were accurate reflections of the AA community’s experience. This was of significance for the group as this influenced their acceptability of the app:

I liked that it was representative of the community that we were...that I’m in. I always look for that in things like apps or magazines...anything that’s print or digital media, I always look for am I represented or reflected in what’s being presented to me. I think that was huge. That was very positive.

Focus group 2, participant

A concern surfaced from a male participant regarding the placement of wording over one of the AA men depicted on the home page visual. The man had a darker skin tone/complexion than the other individuals, and it was felt that this was offensive and, in a sense, reinforced the societal discrimination against and ostracism toward AA men. An AA man in the group exclaimed:

I just wish I could see the dark-skinned brother up there (laughs). I just had a problem with the writing on the brother’s face... I wanted to see his face, and I can’t see it, and it’s like, okay...it would stick out, like every time I opened that app, it bugged me, that’s all.

Focus group 1, participant

### Theme 4: App Implementation

#### Facilitators for Use

Participants identified several facilitators of their engagement with the app features. They placed emphasis on the education modules and their utility for app use.

##### Positive Messaging Supported Engagement

A consistent topic among participants was that the intentional use of positive and encouraging messaging throughout the education modules supported their desire to view the entire series:

They gave us positives even though we’re going through situations, they said “if you just make one change”, so it was encouraging. I liked how they encouraged us to make a small change.

Focus group 2, participant

##### Focus on Benefits of Healthy Lifestyles

The app as a whole increased awareness about and facilitated healthy behaviors among the participants. Participants reported that having this overarching focus infused into the app motivated them to become more physically active and to make healthier food choices:

It definitely made me more aware. I now do 10,000 steps a day…try to get in more vegetables; I don’t always meet that goal to the vegetables, but I do get the fruit in cuz I like those little mandarins; I eat about four or five of those a day (laughs). But it definitely made me more aware of a lot of things; the tracking of the things that we do just made me so much more aware.

Focus group 1, participant

Cuz at one time, I thought, ooh, I walked 2000 steps today; I walked a whole mile (laughs) right ... you sound like me; if you walked in a store, it would be like, ooh, that was a walk (laughs). And now, I’m like…10,000 steps, oh my goodness; oh look at me!

Focus group 1, participant

##### Simple Navigation

Furthermore, a simple navigation layout on the app including a homepage and clear tabs (for tracking and the sharing board) was key to using the app features. Participants noted that it was easy to follow the education module curriculum on a weekly scheduled basis:

Navigation-wise it was very simple. So one key thing that seemed to work for me is if I was like in a particular area and I wasn’t sure where I was going to go, I could look at the top and say, oh, tracking, sharing, you know, should I go back to the homepage. You know, if I was in a module and I wanted to switch to tracking, those links were, to me, they seemed to be available no matter where I clicked. So navigating, I could always get back and forth to the main page where I was...

Focus group 1, participant

##### Visual Display of the Education Module Progress

This was further reinforced by the inclusion of checkmarks to show completion of the education modules as detailed by one participant:

... I also liked the ability to say that you’ve completed the module by selecting “done”. Once you clicked “done,” there was a checkmark there, so it already told you, yeah, you did it...you don’t remember—did I already look at that (laughs)?

Focus group 1, participant

##### Education Module Variety of Activities Appealed to Differing Learning Styles

As a reflection of the initial development of the education module content, participants appreciated the variety of activities within the modules (videos and quizzes) and that they appealed to different learning styles:

I loved the way it was set up because not everybody has a long memory like me. I had to do just a little bit at a time. I thought the design was great because, I mean, you do the pre-quiz, watch the videos and learn... and then do the post-quiz, and that’s a summary of that module. The objective was to learn more about heart health, so I liked having them.

Focus group 1, participant

The education modules and their central feature, videos were viewed as essential app components, as they facilitated learning by the user. One participant shared a positive comment about the succinct “to the point” nature of the modules:

Short and sweet but direct to the point. I thought the videos really made the app...to be honest. I mean I thought that because I’m a visual person; so having someone talk to you without you having to read a book of information was convenient. And they were easier than I was thinking they were going to be (laughs).

Focus group 1, participant

##### Overview of CV Risk Factors at the Start of Education Modules

The order of the education modules was of importance as participants felt that having the “Introduction to Risk Factors” module first among the series of education modules was key to setting the stage for the purpose of the intervention, which in turn promoted continued engagement:

I thought risk factors was important to be first cuz... to me, it engaged me into the application itself. It’s like that first exposure you have to something; you know, when you’re testing out a new app or you’re looking at something new, if you don’t have a good impression on that first one...you might be a little jaded about it...I don’t really want to go back to it...it opens your eyes right away. I thought that having that be one of the first things was engaging, so then I wanted to learn more.

Focus group 1, participant

##### Consolidation of CV Health Information Through Mobile Technology

It was also advantageous to consolidate CV health information from a trusted source on the app over having to search the internet, which was seemingly overwhelming:

...pooling all the information together in one spot because I know that you could probably search the web and find all this information. I thought that was just awesome because they talked about so many aspects of things that can cause heart problems and it was just all in one spot versus if you have to go out and find this information, you might be in several different websites. So I thought that was great.

Focus group 1, participant

You may see these things or hear about it on commercials and stuff but just to be able to have something in your hand and, you know, the world today is all tech-savvy.

Focus group 2, participant

#### Barriers to Use

Participants identified several app features that presented barriers to app usage.

##### Cumbersome Data Entry and Log-In Process

One of the most commonly mentioned barriers was the cumbersome nature of the self-monitoring/tracking feature, which required manual data entry of daily PA and diet patterns by the user. The app’s lack of autosave for the tracking feature presented the greatest challenge. Participants also commented on the multi-step log-in process required by the user to access the app. Although this was intended as a one-time requirement for users at the first long-in, oftentimes, users were required to repeat this process as a security measure or when the user was idle or using different internet networks.

##### Inability to Download Apps on Other Personal Mobile Devices

Participants were provided with tablet devices for use throughout the intervention phase of the study, which had a direct link to the web-based app. As participants enjoyed having the device available through mobile technology, they also found the inability to download the app on other personal mobile devices as a barrier:

The downside was I couldn’t really download anything cuz it was on the app, the iPad that you guys gave us...cuz I wasn’t able to keep the material or print it off because it wasn’t available for me on my own personal iPad.

Focus group 2, participant

### Theme 5: App Impact

The impact of the app on participants’ personal healthy lifestyle change and community was intensively discussed.

#### Positive Changes to Dietary Patterns

Accordingly, participants mentioned specific manners by which the app incited positive changes to their individual dietary patterns:

I think about this even today the fruit and the vegetable intake, I really am more conscientious of that and even when I go to the store, I find myself buying more. I go to the fruit and the vegetables, and my cart seems to have more of that in it than it did before.

Focus group 2, participant

#### Better Awareness of Long-Term Benefits and Motivation to Make Healthy Lifestyle Changes

Participants also highlighted how the app provided them with a better awareness of the long-term benefits of maintaining a healthy lifestyle. They expressed how they viewed the information provided by the app as vital and practical to apply within their daily lives:

...if I continue this way, I’m going to reap better benefits but that information is so vital that you have that information going into the grocery store that this is what I need to buy. Yes, that’s cheaper and I’d get more of that, but this is much healthier and it’s going to reap better benefits for me in the long run.

Focus group 2, participant

Similarly, participants positively expressed that the app provided them with motivation to start healthy lifestyle changes:

...once I started focusing in and how this program showed me just to make slight changes....I started changing my eating habits before I even started going to the gym, and I started losing before I even did anything at the gym.

Focus group 2, participant

#### Team-Based Lifestyle Changes Among Couples and Across Generations

Several participants shared how they noticed that there were several team-based lifestyle changes among couples engaged with the app:

... having a spouse doing it along with them, I think that really encourages because both of those couples were really engaged in terms of the physical activity. I think the one couple...tracked the most steps...over a million steps.

Focus group 1, participant

One participant shared the details of how he and his wife were integrating healthy behaviors as a team:

Me and my wife would go out to dinner; we would get one plate and share it. We would get one entrée, one thing, one meal, and we would cut it in half; we’d tell them to bring us two plates. So we started doing things like that, and that’s what we still do now. So that helps out, and it saves money...

Focus group 2, participant

One of the cascading effects noted from the app was how it inspired an intergenerational healthy lifestyle change among family members. A sense of role modeling of healthy behaviors was observed by younger generations and provoked a desire among them to adopt the same behaviors:

I had to get a youth membership for one of my grandsons because I was going, and he wanted to go along with, and I only have so many guest passes, and he used them all up; so then I had to get a Y membership for him (laughs).

Focus group 2, participant

#### Positive Influence on the Patient-Provider Relationship

Participants identified the positive influence that their participation in the program as a whole had on their relationships with their health care providers. Several participants shared how they were more informed and prepared during their regular check-ups. The information also increased their attention to and understanding of their prior discussions with their health care providers about their CV risk factors. They also were enthusiastically looking forward to sharing their progress toward improving their CV risk factors with their providers:

I told my doctor about it. I took a physical here two weeks ago, and I was telling her, I’m in this Mayo program, in this faith-based program, and it’s wonderful!

Focus group 2, participant

...it made me more knowledgeable about things that I just took for granted and didn’t pay attention to…like my doctor told me… I want you to drop weight. He said, when you come back here next year, you know, just show me some improvement as it is. So when I go back to him, he’s going to like what he sees ‘cause I’ve lost almost 20 pounds. So he’s going to be like, you did good. I liked it because it was something that I took for granted then that I’m really paying more attention to now.

Focus group 2, participant

#### Health Promotion Within the Church Congregation

Another key element that emerged was that the app enhanced health promotion within the church congregation. This was described as communicating the health information learned from the app to weekly worship services with programming for adults and children:

Each Sunday, we bring a health topic to church, and I’ve noticed how people take notes to eat healthy foods.

One other thing we started as a result of this between our Sunday school service and our regular worship service...we have a snack time. We have children that come...they haven’t had breakfast or maybe not even dinner the night before…and then teaching them the importance of eating proper foods, and there are a lot of fruits and vegetables and things like that in there...[Focus group 1, participant]

### Theme 6: Suggestions for App Improvement

Participants had a number of valuable suggestions to improve the app with a focus on certain features.

#### Visuals to See Progress of Diet and PA Self-Monitoring

In particular, participants suggested the use of a visual rewards system or dashboard summary within the app self-monitoring and tracking system to allow users to see their personal progress as it relates to their diet and PA. One participant provided a detailed suggestion:

With my Fitbit, when I reach a goal, I get this little MEEEEEEEE, and it’s like that ... explosion of celebration and it would be nice if, on here, when you put in your fruits and veggies, if you’ve made your five that there was something, like maybe a little star. I mean, it’s more of a visual that—hey, I’m on track; I’m doing good!

...on the calendar that it would show...an apple for the fruit or a banana or something. Some visual that you did it that day. You made it that day, yes.

Focus group 2, participant

Another participant highlighted the need for a weekly summary of diet and PA to increase app engagement by providing the user with useful information:

...because the tracking is great, but if you had a summary for the week, how many fruits and vegetables I’ve eaten, or how many steps have I done for this week...So the more productive the app is, I think the more people will jump on it and want to use it.

Focus group 1, participant

#### Automatic Syncing Function From Other Diet and PA Apps

Participants also suggested integrating an automatic syncing function from PA monitors to upload tracking data directly to the app throughout the day. Participants stressed the inconvenience of having to manually input tracking data and the requirement to complete this “all at once.” Syncing with other diet and PA apps at any preferred time during the day would alleviate the need to have to recall your routine and specific patterns at the end of the day. To illustrate this suggestion, one participant shared:

So if there were integrated apps... say for instance, you’re using some really popular app that allows you to track your calories and your food and stuff...if the faith app could pull information from another app or take that as input without you having to select it over here and go tuck it in there.

Focus group 1, participant

#### Additional Functions for Education Modules and Sharing Boards

Suggestions for additional content to the educational modules included topics related to genetics and CV disease risk. Participants also suggested the inclusion of closed-captioning for viewing content within the education modules, which would also allow them to view videos without sound in the appropriate setting. This feature would also support the needs of those individuals with hearing impairment:

I like seeing what they’re actually saying and put into like it’s a talk or into words or whatever...a closed-caption. Cuz I know sometimes if I’m at work, I don’t have the volume all the way up or something.

Focus group 1, participant

Participants indicated their intention to revisit the education modules and sharing board as a means to reinforce concepts learned from the presenters and to review insights posted from their peers. To accomplish this goal, a search function for topics within features was suggested:

I’d be having conversations with someone, and I couldn’t go back, and I couldn’t find it. The keyword search...that seems like the way to do it. Cuz there were some pretty interesting threads in there too.

Focus group 1, participant

#### Individual Tailoring of the App to Encourage App Use and Increase its Relevance

Participants also suggested that personally tailored reminders (via email or text messages) be sent to participants when there was a lapse in use of the app but not necessarily to those actively engaging with the app. This would help minimize the need for automated email reminders:

...a little reminder that, hey you haven’t cleared your module or you haven’t looked at this new content that we have out there...would be good, but that would be intuitive of the app itself and tailored for the person using it.

Focus group 1, participant

It was suggested that the app allowed participants to configure whether they preferred email or text messages and to include an “on/off” setting.

Finally, participants suggested individual tailoring of the app to specific CV risk factors and for women’s health. One suggested delivery modality included succinct, personal messages to users outside of the education modules related to major CV risk factors such as hypertension or diabetes. One participant discussed this in the context of heart attack warning signs in women:

... even for heart attacks, women—their symptoms are totally different than men, so maybe having a module about that women, certain ages, watch for certain signs cuz this could be a heart attack for you, where in men it’s...and I know a lot of times when you go to the ER, if you present certain symptoms, they don’t think about a woman having a heart attack.

Focus group 2, participant

## Discussion

### Principal Findings

In this formative evaluation, the FAITH! App was perceived as a culturally relevant and acceptable delivery modality for promoting and facilitating positive CV health behaviors for CV disease prevention among AA adults. The main themes that emerged were that the intervention successfully prompted healthy behavioral change through cultural tailoring, multimedia education modules, and social networking. Challenges to healthy behavioral changes related to the intervention primarily revolved around technical malfunctions with the diet and PA self-monitoring features. This formative evaluation contributes to a small but growing body of the literature providing evidence to support mHealth lifestyle interventions to foster sustained behavior change. Taken together, our results provide support for the willingness and eagerness of AAs for the use of mobile devices for health promotion.

A preliminary finding of our formative evaluation was the importance of considering unique cultural influences, as these can be facilitators/barriers to intervention engagement. Few mHealth interventions have adopted tailoring strategies to include content specifically optimized to the sociocharacteristics of AAs [30]. Our parent study is the first to document the development of an mHealth app with faith-based content for CV health promotion to the AA faith community [18]. As maintenance of a healthy religious/spiritual life is central to our prioritized audience [38], our connection of faith and healthy lifestyle through spiritual messaging and biblical scripture was harmonious. It has been previously shown that positive messaging through scripture may heighten the acceptability and use of mHealth interventions by AAs [39]. Our cultural tailoring strategies through both superficial (visual imagery of AAs) and deep (the Black Church) structures also likely increased the receptivity to adapting positive behavioral change strategies [40]. Within our cohort, this was concretely demonstrated by participants’ reports of the organization of potluck events within the churches to sample healthy recipes. This has strong implications in activating change in cultural norms within the Black Church around unhealthy eating [41], thereby improving major CV risk factors such as obesity and diabetes, which are highly prevalent within this group [42].

This study adds to previous work showing the importance of understanding how behavioral theory constructs incite behavioral change in mHealth interventions [8,43,44]. Few studies have similarly explored these behavioral pathways through qualitative approaches. CareSmarts, a theory-based, mobile phone–based intervention was associated with improvements in diabetes self-management among AAs through self-efficacy, social support, and health beliefs [44]. Similarly, our participants’ expressions of healthy lifestyle change supported our underlying theory-driven intervention development model. In line with the social construct theory model, participants were encouraged by a sense of connectedness or commitment to a group that inspired their adherence to positive health behaviors [45]. Several participants compared themselves with others through their interactions on the sharing board, which increased and maintained their motivation toward healthy lifestyle change. Interestingly, participant commentary also suggested that diet and PA behaviors were learned and reinforced in the context of the family unit (spouses and grandparents to grandchildren) [46]. In accordance with earlier studies, this influence of their behaviors on family members is reflective of the family model of reciprocal determinism [47-49]. Furthermore, participants within our study reported that their health beliefs were shaped by the content of the interactive education modules, which in turn enlightened them on their perceived CV risk. This likely further spurred behavior change.

Participants provided several suggestions for improvement to the app that could further strengthen health behavior change. Among these were streamlining the app self-monitoring features, prompts to encourage app use, and personalization based on an individual’s CV risk. On the basis of this valuable feedback, there are plans to integrate these suggestions into the next iteration of the FAITH! App and make the intervention accessible on smartphones. Participants’ feedback also suggests that efficacious approaches should aim to strike a balance between the need for individualized and collective social support for more meaningful and beneficial user experiences. Similar themes advocating for personalization to the individual’s needs were identified by AA gatekeeper stakeholders involved in the development of an mHealth intervention for patients with hypertension [39]. Consistent with our participants, they emphasized the need to include churches in the launch of mHealth interventions. Although these suggestions are closely aligned with a recent systematic review suggesting that personalization and monitoring are effective behavioral change techniques commonly used in mHealth interventions, none of the 21 studies were focused on the preferences/needs of AA end users [43]. This presents a major challenge in drawing conclusions about the most effective features to incorporate into mHealth interventions. The intrinsic advantages of mHealth interventions are their flexibility for delivery in a wide range of settings and their easy adaptability to content to meet the unique needs of specific populations, even at the individual level. Future work is underway to further refine and investigate these app components for AAs within a larger randomized controlled trial.

### Strengths and Limitations

This study is among only a few community-based studies that have performed a comprehensive evaluation through mixed methods of a mHealth intervention among AA adults. This study is also novel in that it describes the in-depth formative evaluation process of a CBPR study aimed at promoting CV health using app-based technology among the AA faith community. As such, our CBPR model provides access to valuable perspectives from an often overlooked, underresourced group at high risk for CV disease. Furthermore, our approach to health promotion may be used as a model to support the AHA 2030 Impact Goals to improve the population’s CV health and well-being while increasing life span, particularly for AAs [50]. Our qualitative findings align with our previously published quantitative results showing improved CV health behaviors (increased daily fruit and vegetable intake and weekly moderate-intensity physical activity) [26]. Thus, they reinforce the impact that our health promotion strategy had on individuals with the greatest CV disease risk.

Nevertheless, we had a very small sampling (9/50, 18%) of parent study participation in the focus groups, possibly introducing recruitment and selection bias. Furthermore, there is a possibility of overestimation of acceptability of the app by participants due to social desirability bias, or those who did not participate in the focus groups may not have found the app to be satisfactory. We implemented strategies to mitigate social desirability bias through rapport-building techniques, pre-fieldwork training with our data collector, and minimization of power differentials [51]. Finally, our study findings may lack generalizability to other AAs residing in other regions of the United States or to other racial and ethnic minority groups. These limitations must be weighed against the opportunity to utilize this novel approach to health promotion in a community-based cohort of AAs.

### Conclusions

This formative evaluation found that the FAITH! App mHealth lifestyle intervention had high reported satisfaction and impact on the health-promoting behaviors of AAs, thereby improving their overall CV health. These findings reinforce the acceptability of mHealth interventions among AAs. Culturally tailored, community-driven mHealth interventions have the potential to support lifestyle behavioral change for CV disease prevention among AAs. Without attention to upfront participatory design followed by rigorous formative evaluation approaches, we may miss the opportunity for the use of innovative mHealth strategies to address health disparities in marginalized populations.

## Abbreviations

AA: African American
AHA: American Heart Association
CBPR: community-based participatory research
CDC: Centers for Disease Control and Prevention
CTSA: Clinical and Translational Science Awards
CV: cardiovascular
eHEALS: eHealth literacy scale
FAITH!: Fostering African-American Improvement in Total Health
mHealth: mobile health
NCATS: National Center for Advancing Translational Sciences
NIH: National Institutes of Health
PA: physical activity
